# Directed Evolution of Microbial Communities in Fermented Foods: Strategies, Mechanisms, and Challenges

**DOI:** 10.3390/foods14020216

**Published:** 2025-01-12

**Authors:** Zihan Yao, Ting Xie, Hongjie Deng, Shuzhi Xiao, Tao Yang

**Affiliations:** College of Food Science and Engineering, Central South University of Forestry and Technology, Changsha 410004, China

**Keywords:** directed evolution, fermented foods, microbial communities, functional optimization

## Abstract

Directed Evolution of Microbial Communities (DEMC) offers a promising approach to enhance the functional attributes of microbial consortia in fermented foods by mimicking natural selection processes. This review details the application of DEMC in fermented foods, focusing on optimizing community traits to improve both fermentation efficiency and the sensory quality of the final products. We outline the core techniques used in DEMC, including the strategic construction of initial microbial communities, the systematic introduction of stress factors to induce desirable traits, and the use of artificial selection to cultivate superior communities. Additionally, we explore the integration of genomic tools and dynamic community analysis to understand and guide the evolutionary trajectories of these communities. While DEMC shows substantial potential for refining fermented food products, it faces challenges such as maintaining genetic diversity and functional stability of the communities. Looking ahead, the integration of advanced omics technologies and computational modeling is anticipated to significantly enhance the predictability and control of microbial community evolution in food fermentation processes. By systematically improving the selection and management of microbial traits, DEMC serves as a crucial tool for enhancing the quality and consistency of fermented foods, directly contributing to more robust and efficient food production systems.

## 1. Introduction

Traditional fermented foods, due to their unique flavor and nutritional value, play important roles in global food culture [1]. Food fermentation is dependent on microbial communities which influence food flavor and texture and play key roles in nutrient transformation and product stability [2] (Table 1).

Advancements in molecular biology and microbial ecology have significantly enhanced our understanding of these microbial communities. This knowledge has spurred interest in employing both ’bottom-up’ and ’top-down’ strategies to optimize these microbial interactions [16]. The ’bottom-up’ approach in microbial community research involves constructing communities from scratch, often starting with a deep understanding of individual microbial strains and their interactions. This method relies heavily on synthetic biology techniques where communities are engineered to have desired properties through extensive genetic modifications and iterative design–build–test–learn cycles [17]. While powerful, this approach can be time-consuming and requires comprehensive knowledge of the genetic and metabolic profiles of all community members, which is not always feasible for complex ecosystems such as those found in fermented foods.

However, microbial community optimization through traditional methods also faces inherent challenges. Natural variability within microbial communities can lead to inconsistent performance, particularly in fermentation processes where minor changes in microbial interactions can significantly affect the final product. For instance, the spontaneous fermentation method often suffers from uncontrolled microbial dynamics, making it difficult to ensure uniformity in quality and functionality [18]. Moreover, the reliance on empirical approaches to manage microbial communities often results in unpredictable outcomes and requires extensive trial and error, limiting scalability in industrial applications. Lastly, maintaining the stability of microbial communities over successive fermentation cycles remains a challenge due to environmental fluctuations and the accumulation of undesired mutations, which can lead to loss of functionality [19].

In contrast, DEMC employs a ‘top-down’ strategy that harnesses the principles of natural selection to evolve microbial communities toward desired functional outcomes. This method simplifies the optimization process by applying selective pressures to steer the natural evolutionary pathways of microbial communities, thereby enhancing their functionality and stability. DEMC offers several advantages over the ’bottom-up’ strategy. First, it focuses on optimizing the functionality and stability of the entire microbial community, without the necessity of decoding complex interactions, making it more suitable for solving complex problems in practical applications [20]. Through the analysis of evolved communities, DEMC can generate new structural–phenotypic associations, assisting in the elucidation of the complex interactions and adaptive evolutionary mechanisms associated with microbial communities. For example, in Chinese liquor fermentation, seasonal temperature variations were identified as the primary factor driving significant changes in microbial community structure. These structural shifts, particularly among bacterial and fungal populations, resulted in functional changes in the metabolome, including variations in key metabolites produced by dominant taxa [21]. Second, DEMC relies on spontaneous mutations rather than genetic engineering. This approach is especially critical in the food industry where food safety is paramount. By avoiding the introduction of genetically modified organisms into fermentation processes, DEMC ensures that fermented products remain natural and safe for consumption, aligning with increasing consumer demands for food safety and transparency in food production [22]. Third, DEMC has demonstrated that applying selective pressures tailored to specific traits can significantly enhance microbial community functionality and stability. For example, in a thermophilic microbial consortium subjected to acid stress, the community developed improved acid resistance and metabolic performance under extreme pH conditions. Such approaches illustrate how ecological adaptation through directed evolution produces microbial consortia that are robust and maintain their functionality, contrasting with synthetic communities engineered through traditional methods, which often lose stability due to ecological interactions and rapid evolutionary changes [23].

Traditional fermented foods face persistent challenges in achieving consistent product quality, efficient production timelines, and resilience to environmental fluctuations [24]. For example, in Chinese liquor fermentation, dynamic microbial interactions and environmental factors like fluctuating acidity, moisture, and temperature often lead to unstable outcomes [25]. Ensuring uniformity in flavor, aroma, and texture across batches remains difficult due to the natural variability within microbial communities, leading to inconsistencies in sensory attributes. For example, subtle differences in yeast strains used for beer brewing can significantly influence the flavor and alcohol content of the beer [26]. DEMC presents a potential solution by selecting microbial consortia that perform reliably under specific fermentation conditions, thereby reducing inherent variability and supporting more consistent outcomes. For example, in syngas-converting microbial consortia, directed evolution under controlled pH conditions enriched species with high ethanologenic potential, achieving up to 72.4% of the theoretical ethanol yield and demonstrating improved stability and consistency [27]. Another challenge is the lengthy fermentation times required to achieve desired qualities, which can decrease production efficiency and increase susceptibility to contamination over extended periods. Traditional soy sauce fermentation usually takes several months to achieve the desired flavor and color [28]. DEMC offers the potential to address this by selecting strains optimized for faster substrate conversion and metabolic efficiency, allowing for reduced fermentation times without compromising on quality. For example, adaptive laboratory evolution combined with mutagenesis was used to enhance the salt tolerance of aroma-producing yeast strains in high-salt soy sauce fermentation. These evolved strains demonstrated increased substrate conversion efficiency and higher volatile compound production, significantly reducing fermentation times while maintaining the desired sensory attributes [29]. Environmental sensitivity is also a major hurdle, as fluctuations in temperature, humidity, and other factors can disrupt microbial activity and lead to product inconsistencies. Temperature fluctuations may destabilize yeast metabolism during beer fermentation, affecting both the taste and alcohol content of the beer [30]. DEMC may improve resilience by selecting for strains with greater tolerance to environmental variations, helping to establish microbial consortia that maintain stability even under variable conditions. For instance, high-pressure acclimation increased the pressure resistance of *Latilactobacillus curvatus* by 1222 times and improved its fermentation performance, including glucose utilization and lactic acid production, by 9% [31].

DEMC aims to improve the sensory quality and fermentation efficiency of traditional fermented products, enhance the stability and resistance of microbial communities to disturbances, and provide solutions to key production challenges. This article discusses the strategies and mechanisms employed in the implementation of DEMC in fermented foods, particularly focusing on the introduction of stress factors that optimize microbial community functions and assessing both the short-term and long-term effects of these interventions. Moreover, it explores the potential and limitations of this method in practical applications, as well as future prospects, offering insights into how DEMC could transform traditional fermentation techniques to meet modern production demands and safety standards.

## 2. Methods

To conduct this comprehensive review, a systematic search was performed across multiple databases, including ScienceDirect, Google Scholar, PubMed, Web of Science, and Scopus. Completed in August 2024, the search targeted studies published between 2000 and 2024. Studies were included if they were published in English and explored DEMC or broader microbial optimization strategies in food fermentation. These strategies focused on enhancing microbial functionality, understanding community stability mechanisms, and improving fermentation efficiency and product quality. Studies unrelated to these themes, outside the timeframe, or published in non-English languages were excluded.

Keywords such as “directed evolution”, “microbial communities”, “fermented foods”, “horizontal gene transfer”, “genetic mutations”, “adaptive laboratory evolution”, “artificial selection in microbes”, and “stress factors in microbial selection” were used individually and in combination. Data from selected studies were categorized into thematic areas, including strategies, mechanisms, and applications of DEMC, and synthesized to extract key insights and trends.

This review has inherent limitations, including its focus on English-language articles and the 2000–2024 timeframe, which may exclude earlier or non-English studies. Despite these constraints, it offers a structured synthesis of recent advancements in directed evolution and microbial optimization strategies, highlighting mechanisms, strategies, and challenges. By providing a balanced overview of current research, it serves as a valuable reference for understanding the potential and limitations of these approaches in fermented food production.

## 3. Strategies for DEMC

**Figure 1:**
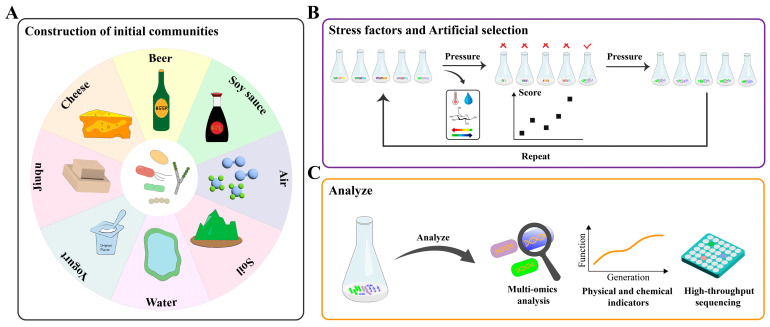
Strategies for DEMC in fermented foods. (**A**) Construction of initial microbial communities: Microbial communities are assembled from various natural and fermentation-related sources, providing the starting point for directed evolution. (**B**) Stress factors and artificial selection: The iterative application of stress factors guides adaptive evolution, with communities selected and refined based on targeted functional traits. (**C**) Functional evaluation and analysis: Optimized communities are analyzed through advanced techniques to evaluate and enhance their functional performance.

In the DEMC approach, researchers first construct an initial microbial community and then induce community responses and functional changes by introducing various stress factors. Subsequently, based on predefined functional indicators, communities that show superior performance are selected for subculturing [32]. This iterative process continues until a stable microbial community that meets specific functional requirements is established (Figure 1).

### 3.1. Construction of Initial Communities

In the application of the DEMC in fermented food production, the construction of the initial microbial community is a crucial step that determines the expression and stability of community functions during subsequent cultivation [24,33] (Figure 1A). Microbial communities in traditional fermented foods typically originate from three main sources: spontaneous fermentation, backslopping, and defined starters. Each method presents unique advantages and limitations that influence microbial diversity, functionality, and product outcomes (Table 2).

In DEMC applications, establishing an optimal initial microbial community involves balancing diversity with targeted functionality. High diversity enhances resilience, adaptability, and growth performance, while allowing specific microbial groups with desirable traits to be optimized over time [18]. However, excessive complexity can lead to interspecies competition that may suppress key functional strains, impacting overall functionality [38]. Conversely, overly simplified communities may lack the robustness needed to adapt to environmental fluctuations. For instance, a study on prokaryotic communities demonstrated that microbial diversity loss impaired specialized ecosystem functions, such as potential nitrification rate, N2-fixation activity, and phosphatase production, particularly in communities with low functional redundancy. In contrast, communities with high functional redundancy were unaffected, highlighting the buffering capacity provided by functional overlap [39]. Therefore, an effective DEMC strategy maintains sufficient diversity to support adaptability while favoring strains that provide the desired functions [40].

### 3.2. Introduction of Stress Factors

In DEMC applications, the introduction of stress factors is essential for steering the functional evolution of the microbial community toward desired traits. The choice of selective pressures used for DEMC should be made based on the desired target. If the goal is to enhance the tolerance of the community to a specific form of environmental stress, such as high temperature or low pH, the direct application of the stressor is usually sufficient. For example, by exposing the community to chemostat selection and evolution at pH 0.75, an evolved consortium comprising *Leptospirillum ferriphilum* (80.32%), *Sulfobacillus thermosulfidooxidans* (15.82%), and *Ferroplasma thermophilum* (3.86%) was developed. This evolved consortium achieved a ferrous iron oxidation rate of 500 mg/L/h and a stable biomass production of 2.0 × 10^8^ cells/mL while maintaining consistent functionality under fluctuating conditions. The consortium also exhibited 55% higher iron extraction efficiency compared to the original community, demonstrating the effectiveness of targeted stress application in driving microbial adaptation and enhancing functional performance under extreme conditions [23]. However, for enhancing complex traits, such as specific flavors that depend on multiple microbial metabolites, selecting appropriate pressures is more nuanced. Research on traditional fermentation processes can reveal key factors influencing microbial community composition and metabolic output. Preliminary experiments may then be used to validate the effectiveness of these factors in guiding microbial community evolution toward the desired flavor profile [41]. Studies have shown that in Baijiu production, long-term exposure to high temperatures fosters specific microbial successions that contribute to the liquor’s rich ester content and complex aroma profile [42].

The methods for applying selective pressures can be classified as progressive or constant. Progressive or incremental pressure involves gradual increases in the pressure intensity, allowing gradual adaptation by the microbes, while constant pressure involves the maintenance of a constant pressure throughout evolution. Both approaches have advantages and disadvantages. Progressive pressure may be better for promoting tolerance, whereas constant pressure may be preferable for selecting microbes that perform best under specific conditions [43,44]. In addition, temporal differences in pressure application may select for different traits; for instance, continuous selective pressures can lead to the selection of stable adaptive traits, whereas intermittent pressures may enhance microbial diversity and resistance to disturbance [45,46].

In traditional food fermentation, controlling environmental conditions is essential to managing the microbial community’s composition and activity. In DEMC, similarly, without careful regulation of stress factors, microbial evolution may deviate from desired functional traits. Thus, selective pressures must be thoughtfully chosen and managed to drive the microbial community toward targeted functionalities, achieving consistency in quality and performance (Table 3).

### 3.3. Artificial Selection

In DEMC, artificial selection is a critical step for cultivating microbial consortia with targeted functional traits. Under carefully selected stress factors, microbial communities often exhibit functional differentiation, allowing for the identification and isolation of microbial groups that display desired functions. These selected groups then serve as the foundation for further iterative culturing under stress conditions, with each cycle of selection refining the community’s traits until they meet the intended functional requirements.

One of the key factors in this process is the real-time monitoring of functional trait markers, such as improved enzyme activity or the production of specific bioactive substances. Omics techniques, such as metabolomics, transcriptomics, and proteomics, are widely used for the experimental evaluation of these goals, allowing for the accurate identification and measurement of key indicators [17,53]. Furthermore, real-time monitoring techniques such as fluorescence quantitative PCR (qPCR) and high-throughput sequencing are used for analyzing microbial community compositions and their dynamic changes. These molecular techniques allow the precise identification and quantification of the presence and abundance of specific functional microorganisms, as well as their changes when adapting to different selective pressures [54,55]. Continuous monitoring allows researchers to track the expression of targeted traits across selection cycles, allowing for timely adjustments in the selection process to optimize trait development [56].

Alongside this, several other factors are essential for successful artificial selection. Genetic diversity within the initial community provides the adaptive potential necessary for functional differentiation under selective pressures. This diversity supports resilience and enables the community to adapt and thrive across multiple generations in changing environments [57]. Maintaining controlled environmental conditions is also critical. Stable parameters, including temperature, pH, and nutrient availability, are vital to avoid fluctuations that could impact community functionality. Precise control over these conditions ensures reproducibility, while aseptic techniques prevent contamination, maintaining the integrity of the experiment [21,25,57]. The intensity of selection also significantly influences the effectiveness of artificial selection. Intense short-term selection pressure can lead to a rapid improvement in community traits, while weaker pressure may be more effective in the long term, as it allows the accumulation of genetic diversity and thereby enhances community adaptability and functional performance [28].

Regarding selection strategies, two common techniques are the propagule and migrant pool methods. The propagule method involves randomly selecting a few individuals from the target community and cultivating them in a new environment to induce new traits in their offspring. The migration pool method, on the other hand, mixes the entire community before selecting individuals for cultivation, potentially reducing genetic diversity due to the initial mixing of all individuals [33]. In a notable application of the propagule strategy, researchers selected bacterial communities for enhanced starch hydrolysis by beginning with a consortium composed of four bacterial strains. Communities were grown, assessed for their starch-degrading capabilities, and the top-performing communities were used to seed subsequent generations. After 17 cycles of selection, these selected communities displayed consistently higher amylolytic activity compared to randomly chosen control communities. This improvement was attributed to the preservation and reinforcement of functional traits within the selected lineages. The microbial communities also demonstrated stability, maintaining their functional capabilities across selection cycles, illustrating the potential of the propagule method for producing functionally robust microbial consortia while minimizing community complexity [58]. Another study used a strategy termed “disassembly selection” to optimize bacterial communities for the degradation of industrial pollutants. Real-time algorithm-guided experiments can explore the combinations of different species in communities, maintaining variability within the communities while enhancing performance [59]. Changing selection strategies can increase the heritability of community traits, thus accelerating evolutionary processes within communities [60,61]. During the implementation of artificial selection, operational details such as the timing of passaging between cultures are also crucial for maintaining efficient community functionality. Studies have demonstrated that early transfers, conducted before nutrient depletion, tend to favor fast-growing microorganisms that rapidly exploit available resources but may overlook slower-adapting strains that contribute to long-term functionality. In contrast, late transfers, performed after nutrient exhaustion, exert stronger selective pressure, resulting in the survival of highly adapted strains. However, this approach increases the risk of population bottlenecks and reduces genetic diversity, which can compromise the overall stability and resilience of the microbial community [28]. Researchers need to continuously optimize passage timing based on community performance to ensure the long-term health and functional expression of the community [62].

Once the bacteria with the expected function are obtained, multiple omics analyses (including genomics, transcriptomics, proteomics, and metabolomics) and strain identification techniques can be used to determine the relationships between microbial community structures and functions [63]. These advanced analyses are able to reveal the complex interactions within microbial communities as well as provide an understanding of the regulatory networks, genetic mutations, and changes in gene expression through the assessment of metabolic pathways and protein expression to achieve specific microbial functions [40,64].

## 4. Mechanisms of DEMC

**Figure 2:**
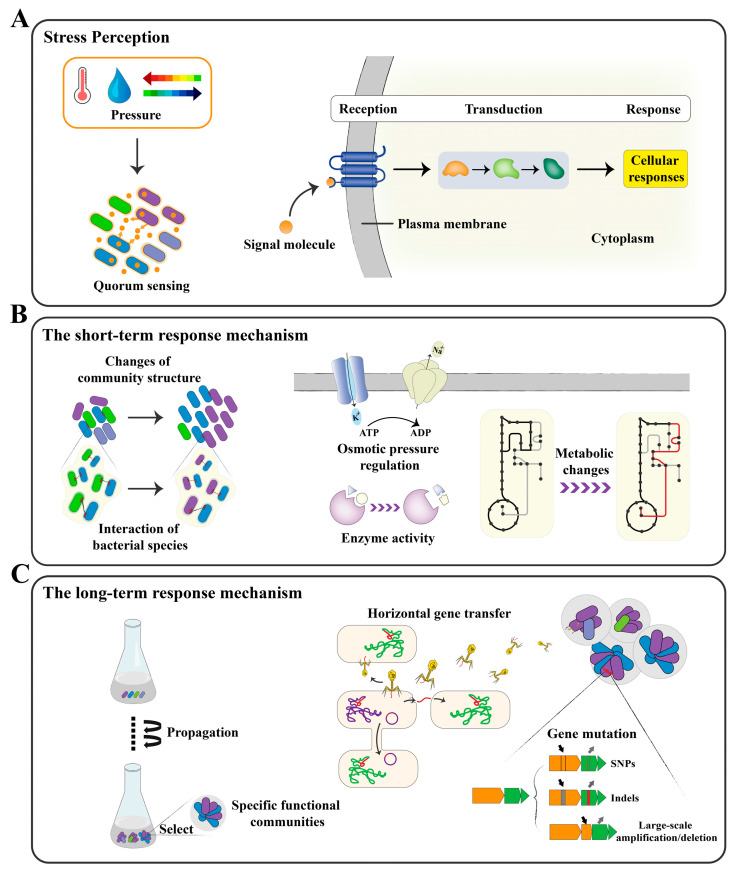
Mechanisms of DEMC. (**A**) Stress perception: QS coordinates community responses, while TCS and GPCRs sense environmental changes and trigger adaptive signaling. (**B**) Short-term responses mechanism: Stress induces shifts in community structure and interactions, while microbes adjust metabolic pathways, enzyme expression, and membrane composition. (**C**) Long-term responses mechanism: Artificial selection drives genetic adaptations through HGT and mutations, enhancing functionality, stability, and resilience.

During the DEMC process, the microbial community shows diverse responses to different stress factors, and, thus, an understanding of the entire process, both in the short and long term, is necessary for the accurate control of the microbial communities [65]. The microbial response to stress factors is a multi-level, dynamic process involving a variety of changes ranging from short-term physiological adjustments to long-term genetic adaptations (Figure 2) [66].

In the short term, microorganisms respond to sudden environmental changes through rapid physiological adjustments, such as alterations in metabolic pathways, modulation of enzyme activities, or changes in metabolite production [67,68]. At the same time, the internal structure of the microbial community and the interactions between community species will also change [69]. These changes may be manifested as increases or decreases in the abundance of certain bacterial species or a reorganization of the interaction networks within the bacterial community [70,71]. These structural adjustments maintain the stability of the community as a whole, enhancing its resistance to environmental stress. During long-term adaptation, heritage microbes may experience genetic changes, including genetic mutations and horizontal gene transfer, through artificial selection, allowing the retention and spread of beneficial genetic variations within the community [72,73]. These genetic changes are often associated with enhanced tolerance to stress and improved efficiency in resource utilization. Such genetic changes often respond to the prolonged presence of environmental stress, ensuring the stability and adaptability of the microbial community within its niche (Table 4) [74].

### 4.1. The Short-Term Mechanism

In DMEC, microbial adaptation to stress begins with the detection of environmental changes through specialized sensors located on cell membranes (Figure 2A). For instance, two-component systems (TCS) in bacteria utilize histidine kinases as sensors and response regulators to facilitate intracellular responses [88]. In fungi and yeast, receptors such as G-protein-coupled receptors (GPCRs) and pathways like the mitogen-activated protein kinase (MAPK) system detect osmotic or oxidative stress [89]. These sensors initiate a cascade of molecular events that regulate genes related to stress response, allowing microbes to adjust to changing conditions. For example, in the HOG pathway in yeast, osmotic stress activates glycerol production to maintain cellular balance, showcasing a classic response to environmental stress [90].

In microbial communities, quorum sensing (QS) enables coordinated responses to external stimuli (Figure 2A). Through the secretion and detection of signaling molecules, QS mediates density-dependent gene expression changes across the community, enhancing collective resilience [91,92]. This process is evident in cocoa bean fermentation, where QS signaling supports biofilm formation and stress resistance, aligning community behaviors to improve functionality in fluctuating conditions [93]. QS and similar signaling mechanisms enable microbial communities to remain adaptable, improving their resilience in the diverse environments typically encountered in fermented food production.

On a micro-level, microbial adaptation to stressors involves changes within individual organisms, such as modifications in metabolic pathways, enzyme activity, and cell membrane composition to enhance survival [94]. For instance, in high-salt environments, microbes increase the synthesis of osmotic regulators such as glycerol and mannitol to adjust the osmotic pressure both inside and outside the cell. This adaptation allows bacteria to accumulate osmotic regulators through metabolic pathway adjustments, thereby enhancing their survival [95,96]. Under low-oxygen conditions, microbes switch to anaerobic metabolic pathways for efficient energy production. In food fermentation, for example, during yogurt production, lactic acid bacteria undergo lactic acid fermentation under low-oxygen conditions, imparting the characteristic sour taste to yogurt [97]. Stress responses also involve altered expression of heat shock proteins and antioxidant enzymes, which protect cell structure and function, helping microbes withstand stress periods. Heat shock proteins prevent protein denaturation at high temperatures, while antioxidant enzymes eliminate free radicals generated by oxidative stress, thus preventing cell damage [98]. For instance, in brewing yeast, the upregulated expression of heat shock proteins improves tolerance to environmental stress during fermentation [68]. Furthermore, microbes alter the fluidity and permeability of cell membranes by adjusting the lipid and protein compositions, thus promoting viability under extreme environmental conditions [99]. In high-temperature environments, bacteria increase the levels of saturated fatty acids to enhance cell membrane stability, whereas in low-temperature environments, the proportion of unsaturated fatty acids is increased to maintain membrane fluidity [100]. These adjustments in physiological characteristics not only improve microbial adaptability but also optimize the functional performance of the microorganisms in food fermentation, thereby enhancing the quality and safety of fermented foods.

On a macro level, these individual adaptive changes drive shifts in the overall community structure, leading to selective pressures that favor specific microbes and functional groups suited to the given environment. Over time, this selection streamlines the community composition, optimizing it for stability and function under targeted conditions (Figure 2B) [101]. For example, salt-tolerant strains are able to perform efficient biotransformation reactions during the fermentation of high-salt products, while microbes with specific enzyme activities play crucial roles in the production of products such as soy sauce and pickled foods by breaking down macromolecules in the raw materials to flavor compounds [5,102]. Through directed evolution, these specific functional microbes can be screened and enhanced to make fermentation processes more efficient while improving the stability and quality of the products.

In addition, changes in community structure during directed evolution involve not only simple dynamics of species gain or loss but also potential new interactions between microbes and the formation of new metabolic pathways [103]. Take, for example, cross-feeding relationships, where one species’ metabolic byproducts become the nutrients for another, thereby linking their survival and growth to one another in a more interdependent manner [104]. In industrial fermentation processes, like ethanol fermentation from sugarcane, interactions between *Lactobacillus amylovorus* and yeast enhance both yeast growth and ethanol production through cross-feeding mechanisms, demonstrating the potential for optimization of synergistic interactions between the microbes [105]. These new biological features can optimize the overall functionality of the community, such as improving energy utilization efficiency, enhancing resistance to environmental stress, and improving biological transformation processes. Synergistic interactions within microbial communities are especially important in the production of fermented foods. For instance, during soy sauce fermentation, proteases and amylases secreted by Aspergillus help break down proteins and starches in the raw materials into amino acids and sugars, which contribute directly to the flavor of soy sauce. Lactic acid bacteria and yeast further metabolize these breakdown products to produce organic acids and aromatic compounds, collectively shaping the complex flavor characteristics of soy sauce [106,107].

### 4.2. The Long-Term Mechanism

During DEMC, genetic variability within microbial populations can be significantly enhanced by introducing specific environmental pressures [108]. These pressures, applied over successive generations, drive long-term changes in microbial communities by selecting for traits that enhance functionality and stability. These variations arise primarily through horizontal gene transfer (HGT) and genetic mutations, both of which accumulate over time, increasing genetic diversity within the population and facilitating adaptation to new environments (Figure 2C). These mechanisms provide the basis for the differentiation and optimization of functional traits, promoting microbial competitiveness and survival capabilities [109,110]. For instance, new genetic variants that are more adapted to the environment may emerge within the population, allowing them to gain competitive advantages and thereby enhancing overall adaptability and survival capabilities [111].

HGT is a pivotal mechanism for microbial adaptation, enabling the exchange of genetic material across species through transformation, transduction, conjugation, and vesiduction [112]. HGT has demonstrated its role in introducing beneficial genes into food fermentation processes [113]. For example, in cheese fermentation, horizontally transferred siderophore genes enhance microbial iron acquisition, improving fermentation efficiency under iron-limited conditions [114]. Similarly, in wine fermentation, oligopeptide transporters acquired through HGT have been linked to improved nitrogen metabolism and enhanced sensory quality of the final product [115].

However, HGT also contributes to the dissemination of antibiotic resistance genes (ARGs) within food fermentation systems, bringing certain risks to food safety and public health. ARGs, such as erm(B) (erythromycin resistance) and tetM (tetracycline resistance), have been identified in Enterococcus faecium strains isolated from fermented sausages and cheese [116]. The spread of these genes through HGT can occur during the fermentation process and may persist in the final product. To minimize these risks, a combination of strategies should be employed, including the use of starter cultures with minimal ARG prevalence, reducing antibiotic contamination in raw materials. Moreover, advances in metagenomics and high-throughput sequencing provide powerful tools for detecting and monitoring ARGs within microbial communities, enabling early intervention when necessary [117].

Genetic mutations further expand microbial adaptability by altering gene structures and metabolic pathways. Unlike HGT, which introduces external genetic material, mutations rely on internal genetic variation and often require multiple generations to accumulate significant adaptive effects. These changes often arise in response to environmental stressors, such as high acidity, salinity, or temperature fluctuations, resulting in enhanced fermentation stability and product quality [111,118]. Through repeated cycles of selection, advantageous genetic traits become fixed, forming robust microbial communities optimized for industrial fermentation. For instance, mutant strains capable of thriving under high-salt conditions have been developed to enhance flavor compound production in fermented products [119].

HGT and genetic mutations collectively enrich the genetic diversity of microbial communities, serving as critical drivers of adaptive evolution in DEMC. These long-term processes allow microbial communities to adapt to diverse environmental pressures over successive generations, enhancing their resilience and functionality in fermentation systems. These mechanisms allow researchers to identify and enhance functional traits that are critical for optimizing fermentation processes. Through repeated selection and reproduction, advantageous genes gradually become fixed in the population, forming an ideal microbial community with stable phenotypes. This process not only maintains the required genetic traits but also enhances the overall functional performance of microbial communities [120].

## 5. Challenges and Future Prospects

Despite its potential, the application of DEMC in fermented foods must address challenges related to the variability and heritability of community functions [33]. Many of these microbial communities have already undergone extensive natural selection during traditional fermentation processes, which can limit the genetic and functional diversity that DEMC relies upon for further improvement [121,122]. This diversity forms the foundation of successful adaptation; without it, the range of possible evolutionary trajectories narrows, reducing the community’s capacity to respond to changing industrial conditions [123,124]. Moreover, artificial selection pressures may inadvertently reduce overall microbial diversity, potentially diminishing the community’s resilience in the face of fluctuating raw material quality and environmental factors [123]. Balancing the need for adequate functional variability with the goal of consistently steering communities toward desired traits thus remains a fundamental challenge.

Ensuring the heritability of community-level traits poses an additional layer of complexity in DEMC. Because the entire microbial consortium, rather than individual strains, is the target of selection, changes in the relative abundance of different species can lead to phenotypic divergence from the intended traits over successive generations [101,125]. Strict control of environmental and operational conditions—such as nutrient composition, pH, or temperature—may help stabilize the desired phenotypes by minimizing random fluctuations and preventing dominance by opportunistic species [126,127]. However, this stability must be weighed against the need to maintain functional diversity. Introducing varied environmental pressures or functional targets during alternating selection cycles can help preserve trait heterogeneity, while the periodic addition of new species or strains can inject fresh genetic and phenotypic diversity into the community [23,128].

The industrial deployment of DEMC also involves complexities and potential risks. Although selection pressures are deliberately applied to enhance specific traits, including flavor or fermentation rate, these conditions can spur unintended microbial evolution, whereby certain subpopulations adapt in unforeseen ways. Over time, these evolutionary pathways may result in the excessive production of certain metabolites, potentially undermining product quality, food safety, or consistency. To mitigate these effects, meta-genomics can be employed to track shifts in microbial community composition, while metabolomics facilitates the quantification of critical metabolic byproducts, thus enabling early detection and targeted intervention. When such measures are integrated into routine quality-control pipelines, these techniques allow researchers to detect and address undesired changes before they significantly affect product integrity. From a regulatory standpoint, the evolved communities, while not produced through traditional genetic engineering, may still be subject to scrutiny if concerns arise about uncharacterized mutations or horizontal gene transfer. Moreover, regulatory frameworks vary widely across regions, influencing the pace at which DEMC strategies can advance from research into practice and shaping the feasibility of their broader implementation. Early engagement with regulatory authorities and adherence to rigorous validation protocols help ensure compliance with diverse guidelines and mitigate safety considerations.

Looking forward, the challenges outlined above underscore the importance of multidisciplinary collaboration to refine and accelerate DEMC strategies in fermented food production. By integrating methods from systems biology, synthetic biology, computational modeling, and engineering, researchers and manufacturers can achieve more predictable and efficient evolutionary outcomes. Digital modeling and machine learning methodologies further enable the targeted design of culture conditions and selection pressures, reducing trial and error in evolutionary cycles. In addition, real-time monitoring tools can facilitate the proactive management of microbial populations, potentially preventing undesirable shifts in community function before they affect product quality. Although hurdles remain, DEMC presents a promising avenue for enhancing fermentation efficiency, sensory quality, and microbial stability. Integrating DEMC with novel bioinformatics tools, high-throughput screening, and advanced computing models may further expedite the discovery of improved microbial consortia, thereby driving innovation in fermented foods.

## Figures and Tables

**Table 1:** Microflora and functional characteristics of fermented foods.

Fermented Foods	Microbial Communities	Main Functions	References
Yogurt	*Lactobacillus delbrueckii* subsp. *bulgaricus*, *Streptococcus thermophilus*	Produces lactic acid, texture improvement	[3]
Sourdough Bread	Lactic acid bacteria (LAB), wild yeasts	Provides unique sour flavor, improves texture	[4]
Kimchi	Lactic acid bacteria (*Leuconostoc* spp., *Lactobacillus* spp., *Weissella* spp.)	Enhances preservation, adds flavor, health benefits	[5]
Natto	*Bacillus subtilis* natto	Produces nattokinase, enhances soybean digestibility	[4]
Vinegar	*Acetobacter aceti*, *Acetobacter pasteurianus*	Produces acetic acid, imparts sour flavor	[6]
Wine	*Saccharomyces cerevisiae*, *Oenococcus oeni*	Alcoholic fermentation, enhances flavor and aroma	[7]
Beer	*Saccharomyces cerevisiae*, Lactic acid bacteria	Alcohol production, contributes to flavor formation	[8]
Kombucha	SCOBY (Symbiotic Culture Of Bacteria and Yeast), including *Gluconacetobacter* xylinus	Produces organic acids, alcohol, and gasses, health benefits	[9]
Baijiu	*Saccharomycopsis*, *Aspergillus* spp., Lactic acid bacteria	Alcohol production, develops complex flavor profiles	[10]
Kefir	*Lactobacillus kefiranofaciens*, *Saccharomyces cerevisiae*, *Dipodascaceae* family yeasts	Contributes to aroma formation and unique flavor profile	[11]
Pickles	Lactic acid bacteria (*Lactobacillus plantarum*, *Lactobacillus pentosus*), *Weissella*, *Enterobacteriaceae*	Enhances acid production (lactic and malic acids), enriches flavor through esters	[12]
Fermented Fruits	Diverse bacterial genera (*Sphingomonas* spp., *Acinetobacter* spp.)	Ensures food safety through microbial diversity, inhibits pathogens	[13]
Fermented Dairy Products	Lactic acid bacteria, *Streptococcus thermophilus*, *Lactococcus lactis*	Improves gut microbiome, provides antioxidant benefits	[14]
Soy Sauce	*Tetragenococcus halophilus*, *Zygosaccharomyces rouxii*, *Candida versatilis*, *Weissella*, *Bacillus*	Microbial succession enhances complex flavor profiles, acidifies the mash, and improves aroma formation	[15]

**Table 2:** Sources of microbial communities in traditional fermented foods.

Methods	Advantages	Limitations	Examples	References
Spontaneous Fermentation	High microbial diversity; contributes to complex flavors and resilience	Variability in outcomes; potential safety issues	Kimchi, sauerkraut, sourdough	[18,34,35]
Backslopping	Continuity in microbial composition and flavor; suitable for maintaining batch consistency	Reduced diversity over time; limits resilience and flavor complexity	Sourdough, cheese	[36]
Defined Starters	Reliable and standardized results; ensures product safety and uniformity	May lack complex and nuanced flavor profiles due to limited microbial interactions	Beer, wine, yogurt	[37]

**Table 3:** The effects of stress factors on microbial communities in fermented foods.

Stress Factors	Impact and Role	Cases	References
Nutrition	Specific nutrients (e.g., sugars, proteins, and fats) activate distinct metabolic pathways in microbes, influencing the flavor and shelf-life of fermented foods.	Serine promotes the growth of Zygosaccharomyces and ethanol production, impacting the flavor profile of Baijiu	[47]
Temperature	Temperature is a critical factor influencing microbial metabolism and growth rates in fermented foods. It affects microbial community dynamics, metabolic pathways, and the production of flavor compounds, ultimately impacting the quality and safety of the final product.	Temperature dynamics significantly correlate with the quick succession of MT-Daqu microbiota in the first 12 days of fermentation, and sustained bio-heat inhibits most microbes’ growth.	[48]
pH	pH significantly influences the structure and function of microbial communities by modifying enzyme activity and metabolite production.	Lower pH is favorable for the succession of sourdough lactic acid bacteria communities, leading to Lactobacillus dominance in the final stages of fermentation.	[49]
Moisture	Moisture content plays a crucial role in the fermentation process of various foods, affecting microbial growth, metabolic activity, and the overall quality of the fermented product.	Fermenting texturized vegetable proteins (TVPs) at 50% moisture content maintains higher chewiness, hardness, integrity index, and layered structure compared to 40% moisture content.	[50]
Salinity	Salt content affects microbial community structure by inhibiting salt-sensitive microbes and selecting for salt-tolerant species.	6% salt addition in suancai fermentation leads to a higher Lactobacillus abundance and better taste quality.	[51]
Oxygen	Oxygen availability influences microbial metabolic pathways and fermentation products.	Oxygen availability affects yeast growth dynamics in mixed culture fermentations, increasing survival time of *Starmerella bacillaris* and decreasing growth rate of *Saccharomyces cerevisiae* strains.	[52]

**Table 4:** Responses of microbial communities in fermented foods to different stress conditions.

Microbial Communities	Stress Conditions	Experimental Outcomes	Mechanisms	Foods/Fermentation Processes	References
*Saccharomyces cerevisiae*	Salt stress (0.7M NaCl)	The yeast showed improved growth rates and survival under high salt conditions	Genome rearrangement and upregulation of specific stress-responsive genes facilitated osmotic balance and ion homeostasis under saline stress	Bread, soy sauce, wine	[75]
*Saccharomyces cerevisiae*	Ethanol stress (8% *v*/*v* ethanol)	Yeast demonstrated increased ethanol tolerance, leading to improved fermentation efficiency	Reconfiguration of metabolic pathways and upregulation of genes involved in ethanol detoxification and membrane stabilization were critical for adaptation	Beer, wine, ethanol production	[76]
*Saccharomyces pastorianus*	Low-temperature stress (<10 °C)	Enhanced fermentation capacity and metabolic efficiency were observed in yeast at low temperatures	Genetic adaptations, including mutations in glycolytic and respiratory pathways, improved energy production efficiency under cold stress	Lager beer, cold brew	[77]
*Lactobacillus* spp.	Salt stress (6% NaCl)	Lactic acid bacteria exhibited increased salt tolerance and lactic acid production	Genomic reorganization and the activation of salt-specific regulatory networks improved ionic balance and osmotic pressure resistance	Pickled vegetables, kimchi, suancai	[78]
*Lactobacillus pentosus*	Acid stress (pH 3.5)	*Lactobacillus pentosus* showed enhanced acid tolerance and metabolic stability	Upregulation of acid stress response genes and modifications of membrane lipid composition enhanced cellular integrity under acidic conditions	Pickled foods, sourdough	[79]
*Saccharomyces cerevisiae*	High-temperature stress (>40 °C)	Yeast showed increased thermotolerance, with improved cell viability at elevated temperatures	Accumulation of heat shock proteins and chaperones, along with alterations in the protein folding and degradation pathways, facilitated thermotolerance	Baijiu, bread, wine	[80]
*Lactobacillus casei*	Lactose stress (high lactose concentration)	*Lactobacillus casei* adapted to high lactose conditions, showing improved growth and lactose metabolism	Genetic mutations in the lactose operon and enhanced expression of β-galactosidase contributed to efficient lactose utilization and adaptation	Yogurt, cheese	[81]
*Lactococcus lactis*	High-temperature stress (>38 °C)	Mutant TM29 grows 33% faster and has a 12% higher lactate production rate at 38 °C than the wild type.	Mutations enhanced thermal tolerance through improved protein expression, membrane synthesis, and gene deletion.	Cheese, sour cream	[82]
*Saccharomyces cerevisiae*	Temperature stress (increased temperature)	Yeast showed improved adaptation to elevated temperatures, enhancing fermentation performance	Genome-wide mutations led to optimized protein quality control and heat shock response, stabilizing cellular functions under thermal stress	Baijiu, beer, wine	[83]
*Clostridium autoethanogenum*	CO_2_ stress (high CO_2_ concentration)	*Clostridium autoethanogenum* showed increased robustness and productivity under high CO_2_ conditions	Genetic adaptations, including modifications in CO_2_ fixation pathways and energy conservation mechanisms, enhanced autotrophic growth and product yield	Biofuel fermentation, ethanol production	[84]
*Saccharomyces cerevisiae*	Alcohol selective pressure (high ethanol concentration)	Yeast strains demonstrated enhanced ethanol tolerance and survival in high-ethanol environments	Mutations in genes related to membrane lipid biosynthesis and stress-responsive pathways contributed to increased membrane integrity and stress resistance	Beer, wine, ethanol production	[85]
*Kluyveromyces lactis*	Ethanol tolerance stress (high ethanol concentration)	*Kluyveromyces* lactis showed enhanced ethanol tolerance under high ethanol conditions	The regulation of ethanol-responsive genes and stabilization of cellular membranes via lipid modification played key roles in adaptation	Wine, ethanol production	[86]
*Lactobacillus casei*	Freezing stress (−20 °C)	*Lactobacillus casei* exhibited improved survival and metabolic stability under prolonged freezing conditions	Upregulation of cold-shock proteins and restructuring of membrane lipids improved cellular resilience to freezing-induced damage	Frozen yogurt, frozen fermented foods	[87]

## Data Availability

No new data were created or analyzed in this study. Data sharing does not apply to this article.
